# c-MYC Expression Is a Possible Keystone in the Colorectal Cancer Resistance to EGFR Inhibitors

**DOI:** 10.3390/cancers12030638

**Published:** 2020-03-10

**Authors:** Antonia Strippoli, Alessandra Cocomazzi, Michele Basso, Tonia Cenci, Riccardo Ricci, Francesco Pierconti, Alessandra Cassano, Vincenzo Fiorentino, Carlo Barone, Emilio Bria, Lucia Ricci-Vitiani, Giampaolo Tortora, Luigi Maria Larocca, Maurizio Martini

**Affiliations:** 1 Fondazione Policlinico Universitario A. Gemelli IRCCS, Largo A. Gemelli 8, 00168 Rome, Italy; strefoantonia@gmail.com (A.S.); michele.basso@policlinicogemelli.it (M.B.); riccardo.ricci@unicatt.it (R.R.); francesco.pierconti@unicatt.it (F.P.); alessandra.cassano@unicatt.it (A.C.); carlo.barone@unicatt.it (C.B.); emilio.bria@unicatt.it (E.B.); giampaolo.tortora@unicatt.it (G.T.); luigimaria.larocca@unicatt.it (L.M.L.); 2 Department of Life Sciences and Public Health, Università Cattolica del Sacro Cuore, Largo F. Vito 1, 00168 Rome, Italy; alessandra.cocomazzi@libero.it (A.C.); tonia.cenci@gmail.com (T.C.); vins8966@gmail.com (V.F.); 3 Department of Translational Medicine and Surgery, Università Cattolica del Sacro Cuore, Largo F. Vito 1, 00168 Rome, Italy; 4 Department of Oncology and Molecular Medicine, Istituto Superiore di Sanità, Viale Regina Elena, 299, 00161 Rome, Italy; lriccivitiani@yahoo.it

**Keywords:** c-MYC, colorectal cancer, EGFR inhibitor resistance, targeted therapy

## Abstract

Alterations in the transcriptional factor c-MYC could be involved in the anti-EGFR resistance in metastatic colorectal cancer (mCRC). The c-MYC expression was evaluated in 121 RAS and BRAF wild-type mCRC before treatment with anti-EGFR+Folfiri therapy and in 33 subsequent metastases collected during target therapy (TT) or in TT resistance phase. We analyzed the expression and the functional role of some c-MYC linked miRNAs (miR-31-3p, miR-143 and miR-145) in our patient group and in two CRC cell lines, also performing a c-MYC target PCR array. Patients with higher c-MYC expression (HME) showed a significant lower PFS and OS when compared to those with low c-MYC expression (LME). HME pattern was significantly more frequent in the metastases after TT and significantly associated to anti-EGFR molecular resistance alterations. We also found a significant correlation between the expression of the above-mentioned c-MYC linked miRNAs, c-MYC level and anti-EGFR resistance. Moreover, expression gene profiling pointed out the pivotal role of c-MYC in CRC-related cell-cycle, apoptosis, signal transduction and cell-growth pathways. c-MYC expression might distinguish patients with a lower PFS and OS in anti-EGFR treated mCRC. The individuation of some miRNAs involved in the c-MYC pathway regulation and the downstream c-MYC effector genes could provide a new possible target to overcome the anti-EGFR resistance in mCRC.

## 1. Introduction

The treatment of metastatic colorectal cancer (mCRC) was deeply changed by the introduction of anti-EGFR “targeted therapy” (TT) [1,2]. However, the EGFR blockage therapy is restricted to about 40% mCRC patients not showing KRAS and NRAS oncogenic mutations, the main TT primary molecular resistance, and only half of these are responsive to the TT treatment [3,4]. Although there are no significant results to support the hypothesis, other molecular alterations such as functional mutations of BRAF, PI3KCA, PTEN, PDGFRA or MEP2K1 and amplifications of c-MET, ERBB2 and FGFR1 genes could likely be involved in the primary-intrinsic resistance to anti-EGFR TT [3,4,5,6]. 

However, after a variable period of response, TT inevitably leads to acquired resistance or secondary resistance [5,6,7]. This can depend on various molecular alterations, mainly caused by activation of compensatory kinases, supporting the idea that deregulation of kinase activity could represent not only the major mechanism by which cancer cells evade normal physiological constraints on growth and survival, but also the principal responsible of the TT resistance, especially under pharmacologic pressure (as the so-called “kinome reprogramming mechanism”) [8,9,10].

Recent reports have demonstrated that divergent kinase signaling pathways frequently converge on common and relatively small downstream effectors [10,11]. These molecules, including transcription factors, could represent a “fragile-point” in the kinases-addicted carcinoma and in the “kinome reprogramming phenomena” observed in TT resistance [12]. In this way, Shen et al. have recently demonstrated that c-MYC would be involved in the response to c-MET inhibitor therapy both in c-MET addicted cancer cell-lines and in derived resistance, including colorectal cell lines [11]. Moreover, numerous authors have reported the significant upregulation of c-MYC protein as a downstream effector of frequently altered kinase MAPK and RAS pathways in CRC, where this transcription factor plays an essential role in tumorigenesis and in chemoresistance to oxaliplatin treatment of colon cancer stem cells. [13]. Additionally, several scientific works have also described the functional effector role of c-MYC downstream of many other oncogenic kinases such as BRAF and EGFR, probably representing the main fulcrum of the primary and secondary resistance in targeted therapy [14]. In this scenario, several studies have recently demonstrated that some miRNAs (miRs), short noncoding RNAs controlling gene expression at posttranscriptional level, are also involved in the resistance to treatment in different human tumors. Among these, clinical and biological data showed that miR-31, miR-143 and miR-145 could have a pivotal role in the resistance to anti-EGFR treatment in mCRC, even being identified as predictive markers of response to therapy [15,16,17,18,19,20].

Here, we analyzed the role of c-MYC pathway in a TT-treated cohort of KRAS, NRAS and BRAF wild-type mCRC patients, and in a subgroup of liver metastasis patients subjected to conversion chemotherapy that led to surgical treatment. Results were correlated with the patients’ clinical characteristics and biological tumor features. We identified specific altered genes and miRNAs, linked to c-MYC pathway, as possible new molecular targets to overcome the anti-EGFR resistance. Moreover, we found that c-MYC is a significant predictive marker of anti-EGFR therapy response in mCRC then representing a possible “fragile point” among the various molecular alterations involved in TT resistance. 

## 2. Results

### 2.1. c-MYC Expression in mCRC and Correlation to the Therapy Response 

**Figure 1:**
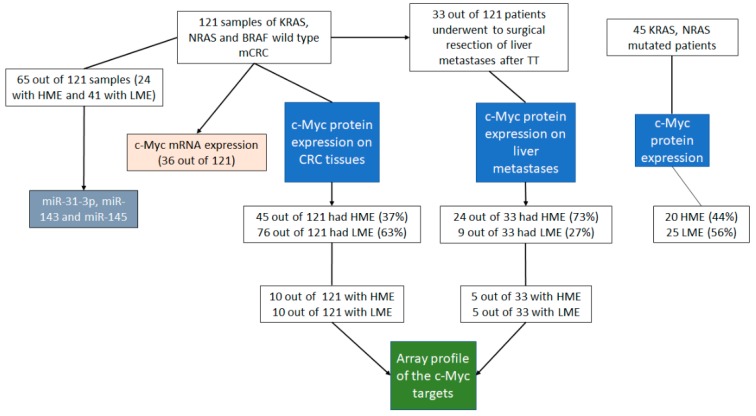
The figure shows the flow chart of the different analyses performed in this work in the different patient subgroups.

**Figure 2:**
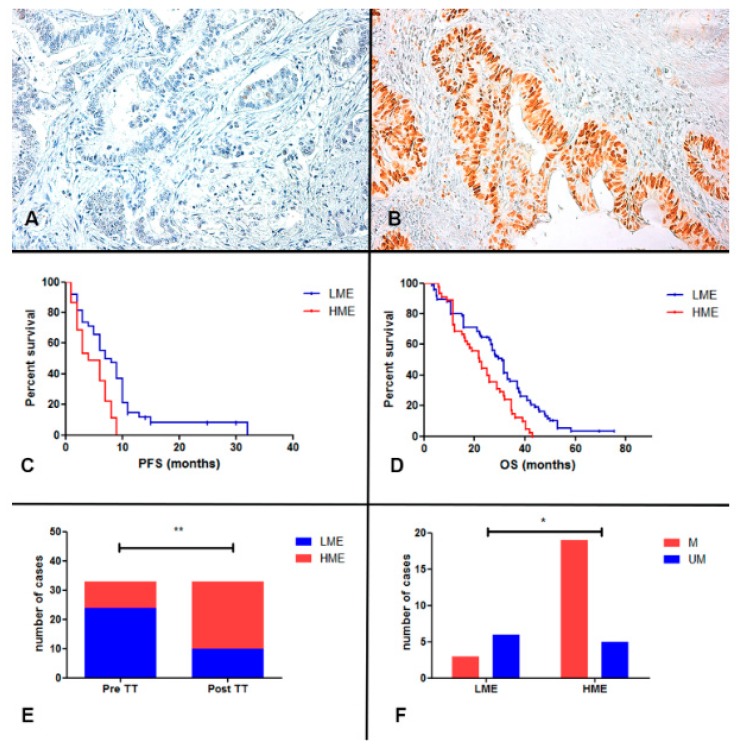
Panel (**A**,**B**). Immunohistochemical analysis of c-MYC protein expression. The figure shows two representative cases of CRC with positive (panel (**B**); score 8) and negative staining (panel (**A**); score 1) for c-MYC (Original magnification 200×); Panel (**C**,**D**). Kaplan-Meier curves for PFS and OS of RAS-BRAF wild-type anti-EGFR mCRC patients stratified by c-MYC expression. LME patients (blue-line) was significantly associated to a better PFS (*p* < 0.0001) and OS (*p* = 0.0016) respect to HME (red-line). Panel (**E**) The figure shows that the HME cases were significantly higher in the metastatic liver samples after TT (post-TT) in comparison to the correspondent primary CRC (Pre-TT; *p* = 0.0012; Fisher’s exact test); Panel (**F**) The HME metastases after TT had a significant higher molecular alterations (M) respect to LME cases (*p* = 0.0334; M vs. UM, liver metastasis without molecular alterations after TT; Fisher’s exact test). * *p* < 0.05, ** *p* < 0.01.

In this retrospective study, we assessed the expression of c-MYC using immunohistochemical staining, in 121 samples of KRAS, NRAS and BRAF wild type mCRC receiving anti-EGFR+Folfiri therapy in the first line treatment. We found a high expression level of c-MYC (HME; score from 4 to 9) in 45 out of 121 (37%) mCRC patients, while 76 (63%) cases showed a low expression of c-MYC (LME; score from 0 to 3; Table 1; Figure 1 and Figure 2 panel A and B). On the contrary, all normal colonic mucosae samples and liver tissue around the metastases showed LME (score 0–3, data not shown). When we correlate the expression of c-MYC with the progression free survival time (PFS) and overall survival (OS), we found that patients with HME had a significant lower PFS and OS than those with reduced c-MYC expression (Table 1; Figure 2C for PFS: median PFS for HME patients 5 months versus median PFS for LME patients 9 months, *p* < 0.0001, HR 2.099, 95% CI from 1.3637 to 3.2320; Figure 2D for OS: median OS for HME patients 22 months versus median OS for LME patients 31 months, *p* = 0.0016, HR 2.0322, 95% CI from 1.3093 to 3.1542).

In order to understand if the expression of the c-MYC bears a predictive and/or prognostic role in mCRC regarding TT resistance, we also analyzed the expression of this gene in 45 KRAS, NRAS mutated patients (Figure 1 and Table S1) that were not eligible for anti-EGFRs containing regimens. These patients were treated with anti-angiogenic drug bevacizumab plus chemotherapy regiment (Table S1). Unlike what observed in patients with mCRC treated with anti-EGFR+Folfiri therapy, the expression of c-MYC did not show any correlation with PFS and OS in the anti-angiogenic plus chemotherapy treated cohort (Figure 1 and Figure S1 panel A for PFS: median PFS for HME patients 5 months versus median PFS for LME patients 6 months, *p* = 0.7159, HR 0.8829, 95% CI from 0.4514 to 1.7268; Figure S1 panel B for OS: median OS for HME patients 25 months versus median OS for LME patients 23 months, *p* = 0.8083, HR 1.0799, 95% CI from 0.5805 to 2.0087). 

Multivariate analysis of PFS, including c-MYC expression, age, liver limited disease (LLD), histological grade and site of primary tumor showed that c-MYC expression was the only significant predictor (*p* = 0.0125, 95% CI: from 14,545 to 209,951; Table S2). Multivariate analysis of OS showed that the independent prognostic variables were LLD and c-MYC expression (*p* = 0.0093, 95% CI: from 1.1453 to 2.5786 for c-MYC expression; *p* = 0.0021, 95% CI: from 0.3241 to 0.7769 for LLD; Table S3).

We also correlated c-MYC protein and the mRNA expression in 36 patients (13 with HME and 23 with LME) founding a nonsignificant association between c-MYC nuclear score expression and c-MYC mRNA level (Spearman r = 0.13; *p* = 0.4289; Figure 1 and Figure S2).

### 2.2. c-MYC Expression in Metastases after Anti-EGFR Inhibitors Therapy

During the TT treatment or in the resistance phase, 33 out of 121 (27%) patients underwent surgical resection of metastases (Table 1 and Figure 1; these cases belong to the 37 metastases confined to liver). Analyzing the c-MYC expression in the liver metastases after TT therapy, we found HME in 24 out of 33 patients (73%), while only nine cases (27%) showed LME. The c-MYC expression was significantly different in the corresponding primary tumor of these cases, with a prevalence of LME patients (n = 23, 70% vs. HME n = 10, 30%; *p* = 0.0012, OR 6.133; 95% CI from 2.110 to 1.83, Fisher’s exact test; Figure 2E). In addition, HME primary tumor maintained the same pattern after TT, whereas 14 LME patients changed in high c-MYC expression after anti-EGFR therapy (decremental percentage of almost 60%). Moreover, analyzing the expression of c-MYC in a small subgroup (10 cases) of metastases before the treatment we found that three had HME and seven LME pattern, showing the same c-MYC expression observed in the corresponding primary tumors.

### 2.3. RAS, BRAF, EGFR Mutations and HER2 and c-MET Amplification in Metastases after Anti-EGFR Inhibitors Therapy

Thirty-three post-TT therapy metastatic samples were investigated for molecular alterations involved in anti-EGFR resistance, including the RAS, BRAF and EGFR mutational assessment and HER2 and c-MET amplification analysis. Out of the 33 samples, 14 (42.4%) showed a RAS mutations, three (9.1%) BRAF mutations, two (6.1%) EGFR mutations, two (6.1%) c-MET amplifications, while one featured a HER2 amplification (3%). Interestingly, when we correlated the amount of molecular alterations with the c-MYC expression, we found that 19 out of 33 cases showed a HME pattern while only three cases showed a LME pattern (Figure 2F; *p* = 0.0334, OR 0.1316, 95% CI from 0.02402 to 0.7208; Fisher’s exact test). All three LME cases showed RAS mutations. Interestingly, we did not find any molecular alterations described above in ten out of 33 metastases before treatment. 

### 2.4. Role of miR-31, miR-143 and miR-145 in the c-MYC Regulation

**Figure 3:**
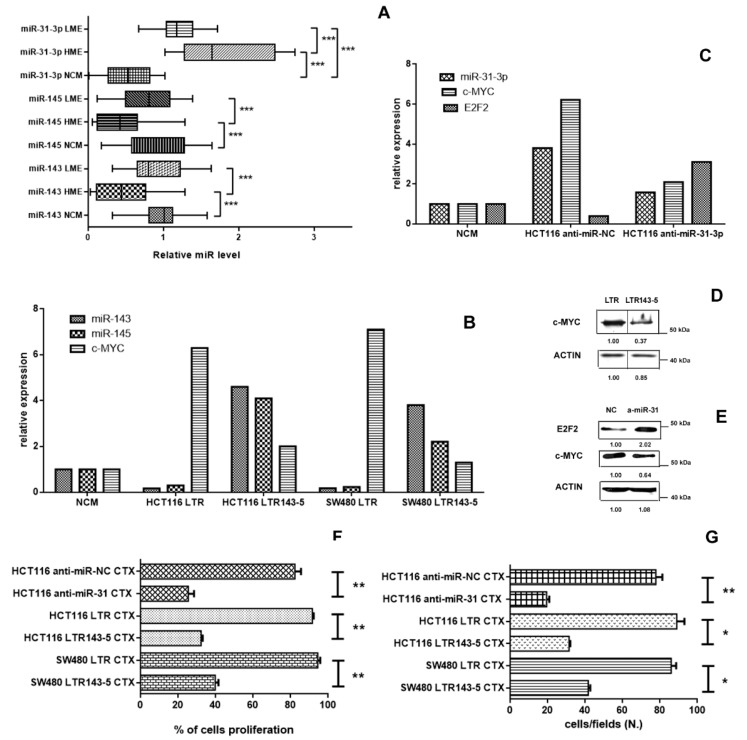
Panel (**A**). The figure shows the significant low expression of miR-143 and miR-145 in the HME cases in comparison to the LME cases (*p* = 0.0001 and *p* = 0.0006, respectively; Mann Whitney t test) and normal colonic mucosa (NCM; *p* = 0.0002 and *p* = 0.0005, respectively; Mann Whitney t test); conversely, miR-31-3p had a high expression both in HME and LME cases respect to the normal colonic mucosa (*p* < 0.0001 and *p* < 0.0001, respectively; Mann Whitney t test). Panel (**B**). MiR-143 and miR-145 expression show low level in HCT116 and SW480 cell lines transduced with LTR, while c-MYC expression was upregulated in both cancer cell lines. Conversely, LTR143-5 transduction cell lines showed an upregulation of the two miRs and a c-MYC downregulation. Panel (**C**) Mir-31-3p and c-MYC expression were downregulated in HCT116 cell lines transduced with anti-miR-31-3p in comparison with the controls, while E2F2 transcription factor was upregulated. Panel (**D**) Western-blot analysis for c-MYC expression in HCT116 cells transduced with LTR and with LTR143-5 (a representative image with, below, the relative expression ratio below). Panel (**E**) Western-blot analysis for c-MYC and E2F2 expression in HCT116 cells transduced with NC and with anti-miR31-3p (a representative image with, below, the relative expression ratio below). Panel (**F**,**G**). The figures show the significant cytotoxicity (panel F) and the significant reduction of the number of migrated cell (panel G) of cetuximab-treated (CTX) LTR143-5-transduced HCT116 and SW480 cell lines and cetuximab-treated (CTX) anti-miR-31-3p HCT116 respect to the those transduced with LTR and NC (*p* < 0.05; Mann Whitney *t* test). * *p* < 0.05, ** *p* < 0.01, *** *p* < 0.001.

In line with recent studies that highlight the central role of some miRNAs in the resistance to therapies of many human tumors, several authors have shown that some of them, such as miR-31, miR-143 and miR-145, are associated with resistance to the anti-EGFR therapy in mCRC [15,16,17,18,19,20]. Therefore, we analyzed the miR-31-3p, miR-143 and miR-145 expression in a subgroup of 65 out of 121 mCRC and in 20 normal colonic mucosa (NCM; Figure 1). The miR-31-3p expression was significantly upregulated in the HME group (24 patients) and LME group (41 patients) with respect to the normal colonic mucosa (NCM, 20 patients; HME vs. NCM, 3-fold, *p* < 0.0001; LME vs. NCM, 2.2- fold, *p* < 0.0001; Figure 3A). In addition, the miR-31-3p expression was also significantly higher in HME patients in comparison to LME patients (HME vs. LME, 1.4-fold, *p* < 0.0001). When we correlated expression of miR-31-3p with the PFS (all 65 analyzed cases), we found that patients with higher level of this miRNA had a significantly lower PFS than those with reduced miR-31-3p expression (Figure S3 panel A for PFS: median PFS for high miR-31-3p expression 5 months versus median PFS for low miR-31-3p expression 9 months, *p* = 0.0012, HR3.5011, 95% CI from 16,418 to 74,662). We interestingly found a significant association between c-MYC nuclear score expression and the expression of this miRNA (Spearman r = 0.69; *p* < 0.0001; Figure S3 panel B).

We also found that miR-143 and miR-145 level was significantly reduced in HME samples (24 patients; 2.31-fold for miR-143 and 3.15-fold for miR-145) respect to the normal colonic mucosae (NCM, 20 patients; *p* = 0.0002 and *p* = 0.0005, respectively; Figure 3A) and LME group (41 patients; *p* = 0.0001 and *p* = 0.0006, respectively; Figure 3A). Conversely, miR-143 and miR-145 did not show a significant reduction (about 1.21-fold for miR-143 and 1.04-fold for miR145) in LME cases with respect to the NCM (*p* = 0.2882 and *p* = 0.1429, respectively; Figure 3A).

To evaluate the effect of miR-31-3p, miR-143 and miR-145 on the c-MYC expression in colorectal cancer, we deregulated these miRs in two anti-EGFR resistant cell lines: KRAS mutated (p.G13D) HCT116 and KRAS mutated (p.G12V) SW480 cell line [21]. Both cell lines have a reduced expression of miR-143 and miR-145 and a c-MYC hyperexpression (Figure 3B), while HCT116 cell line showed a hyperexpression of miR-31-3p (Figure 3C). After miR-143 and miR-145 transfection, we found an about 10-fold upregulation of these two miRs and an about 3-fold reduction of c-MYC expression in these two cell lines compared with those transfected with LTR (Figure 3B,D). Similarly, when we transfected HCT116 cell line with anti-miR-31-3p, the miR-31-3p and c-MYC expression levels decreased by 2.5-fold with respect to those transfected with NC (Figure 3 panel C and E). In addition, the expression of E2F2, a transcription factor identified as a direct target of miR-31 in colorectal cancer [22], increased by 4-fold after transfection of HCT116 cell line with anti-miR-31-3p in comparison to the NC (Figure 3 panel C and E).

Notably, after separation of the nuclear and the cytoplasmic fraction in both cell lines, we found that c-Myc protein expression was higher in nuclear extract in comparison to the cytoplasmic fraction in both cell lines (by an about 3-fold increase) highlighting the significant different expression between the two cellular compartments (data not shown).

When we treated these transfected cell lines (SW480 cell line with miR-143 and miR-145 and HCT116 cell line with miR-143 and miR-145 and anti-miR-31-3p) with cetuximab, we found a significant reduction of the cell proliferation and migration (Figure 3 panel F and G; *p* < 0.05) in comparison to the cell lines transfected with LTR and anti-miR-NC, respectively. According to Pagliuca et al., the upregulation of miR-143 and miR-145 determined the downregulation of RAS-MAPK axis (data not shown) [20].

### 2.5. Altered Genes Expression Profile in the mCRC with HME and LME

To identify the alterations underlying the involvement of c-MYC in the TT resistance, we evaluated the gene expression profiles of the c-MYC targets by microarray analysis (PAHS-177ZD, purchased from Qiagen) in 10 HME cases in comparison to 10 LME mCRC cases (Figure 1).

**Figure 4:**
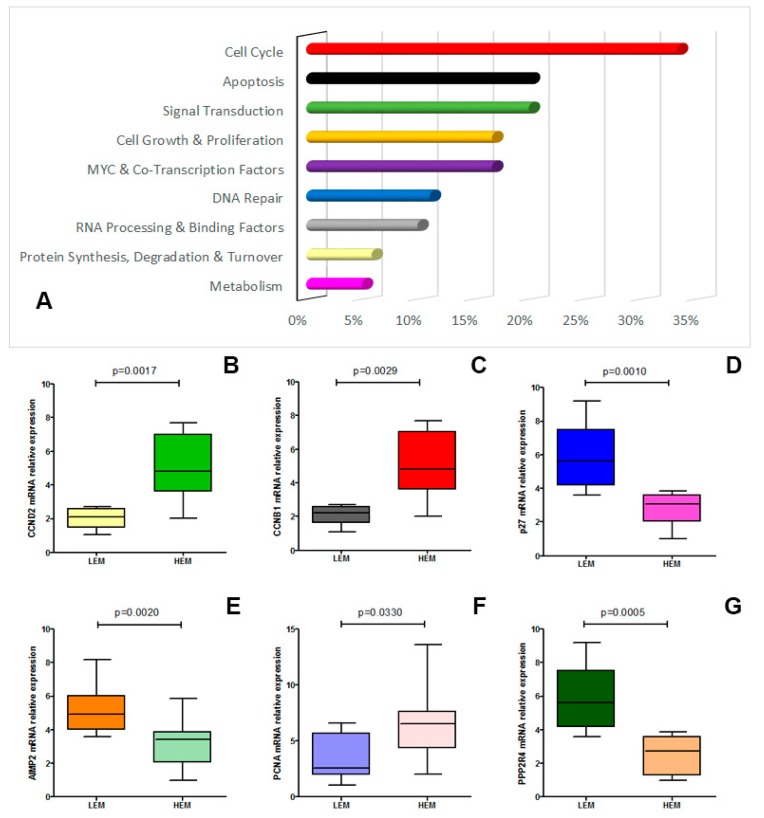
Panel (**A**). The figure shows the effect of c-MYC overexpression on several cellular pathways (expressed as altered genes percentage/pathway); Panel (**B**–**G**). Real-time confirmed expression of altered genes comparing HME and LME patients (CRCND2, *p* = 0.0017; CRCNB1, *p* = 0.0029; p27, *p* = 0.0010; AIMP2, *p* = 0.0020; PCNA, *p* = 0.0330; PPP2R4, *p* = 0.0005).

Expression analysis highlighted that the two groups showed an altered gene expression (Figure 4A). Significant alteration of some genes was confirmed by real-time analysis (Figure 4B–G and Table S4).

We found that the higher number of altered genes belonged to the cell cycle pathway (about 35% of the analyzed genes). In this category CRCNB1, CRCND2 genes were significantly overexpressed (HEM vs. LEM, *p* < 0.01) while CDKN1B (p27KIP1) showed a significant downregulation (HEM vs. LEM, *p* < 0.001).

The second, third and fourth group with the most altered genes belonged to the apoptotic, signal transduction and cell growth and proliferation pathways (about 20% of analyzed genes), while we found only low or few significant alterations between the HME and LME groups regarding the DNA repair, RNA processing and binding factors pathway and of the transcription factors family (about 15% of analyzed genes) and the protein synthesis, degradation and turnover and in the metabolism pathways (about 5% of analyzed genes; Figure 4B–G and Table S4).

When we analyzed the c-MYC pathway in five HME metastases in comparison to five LME metastases, we found the same gene alteration described in the HME and LME CRC groups (Figure 1). Interestingly, LME CRC treated with TT showing HME in the metastatic tissues, also had the same c-MYC target genes expression change (data not shown).

## 3. Discussion

In this work, we have demonstrated that higher c-MYC levels (HME group) in RAS-BRAF wild type mCRC are significantly associated with a higher and faster resistance to anti-EGFR plus chemotherapy treatment in both univariate and multivariate analysis (*p* < 0.0001 and *p* = 0.0125, respectively). Moreover, we found a significant association between HME and lower OS, candidating c-MYC as a possible negative prognosticator in TT treated mCRC. The exclusive predictive and prognostic role of c-MYC expression in anti-EGFR plus chemotherapy treated mCRC was also indirectly confirmed by the not significant association of this transcription factor with PFS and OS in a mCRC cohort receiving anti-angiogenetic drug plus chemotherapy.

The involvement of c-MYC in the anti-EGFR resistance mechanism was also highlighted by the significant increase of HME cases in liver metastases resected after or during TT treatment in comparison to the primary tumor (*p* = 0.0012). This data was functionally reinforced by the significant association between HME and the anti-EGFR resistance molecular alterations, arisen in the liver TT treated metastases (*p* = 0.034).

Although a recent consensus molecular analysis individuated the c-MYC alteration as a specific marker of a particular CRC sub-group (the CMS2 subtype), definitively confirming the involvement of c-MYC in the CRC pathogenesis, the same study did not find a significant association of this group to a better or worse OS or PFS [23,24]. Similarly, several works and meta-analyses of public databases (also including the TCGA database) seem to strengthen the controversial prognostic value of c-MYC in CRC [25]. Probably, the multi-variegate treatments, the methodological analysis of c-MYC expression based on the mRNA levels and the clinical inhomogeneity of the analyzed cohorts could play a role in these results [23,24,25].

Following an initially brilliant response to kinase-targeted therapies, also including anti-EGFR mCRC treatment, mutations (either acquired or selected), able to decrease the receptor affinity for kinase inhibitors or to produce a kinome reprogramming resistance that develops through alternate routes of kinase pathway activation, tend to occur [10,12,13]. Notwithstanding, several scientific reports have recently highlighted that divergent kinases signaling pathways often converge on common downstream effectors, including transcription factor such as c-MYC, that could represent the “Achilles’ heel” of the kinase-addicted cancer, providing important therapeutic implications [10,12,13]. In addition, an increasing number of studies highlighted the role of c-MYC in the anti-kinases therapy resistance in different tumors, also including the anti-EGFR in mCRC. Yu Y. and colleagues demonstrated that FoxO3a, a transcriptional factor with an important regulatory function in the CRC cell survival, confers cetuximab resistance in RAS mutated colorectal cancer cell lines through a c-MYC induced expression [26]. Similarly, Shen et al. recently demonstrated that c-MYC would be involved in the response to c-MET inhibitor therapy both in c-MET addicted cancer cell-lines and in derived resistance [11]. In this scenario, the c-MYC blockade, acting on upstream regulators or downstream on activated genes, in association with other specific anti-kinase inhibitors could circumvent the acquired resistance to anti-EGFR preventing the kinase reprogramming.

Recently, several studies demonstrated that low miR-31-3p expression was significantly associated with improved outcome and prolonged benefit in the anti-EGFR plus chemotherapy treatd mCRC, becoming this miRNA as a probable biomarker for the selection of candidates to this first line treatment [15,16,17]. Here we confirmed the predictive value of miR-31-3p in our cohort (*p* = 0.0012), also demonstrating that the expression of this miRNA is significantly correlated to the c-MYC protein expression. Moreover, with functional experiments, we demonstrated that after inhibition of the miR-31-3p expression in HCT116 cancer cell line, the c-MYC expression considerably decreased, probably through a direct action of this miRNA on E2F2, a negative regulator of the c-MYC expression [22]. Although several scientific works, including ours, have confirmed the predictive role of miR-31 in resistance to anti-EGFR treatment in mCRC, to date, the molecular mechanisms underlying this resistance are unclear [16]. In this work, we investigated one of these possible mechanisms, showing that E2F2 could have a role not only in being a direct effector of miR-31 in the regulation of the colorectal cancer proliferation [22], but also in the resistance to treatment with anti-EGFR.

Similarly, other publications demonstrated a functional connection between miRNA-143 and miRNA-145, EGFR pathway and c-MYC. EGFR activation downregulated these two miRs which in turn determine the upregulation of KRAS and c-MYC [18,19,20]. We demonstrated that patients with HME had a significant reduced miRNA-143 and miRNA-145 expression (*p* = 0.0002 and *p* = 0.0005, respectively). In addition, using two mutated KRAS CRC cell lines, we found that the upregulation of these two miRs were able to downregulate the expression of c-MYC. Several scientific reports have shown that miRNA-143 and miRNA-145 are tumor suppressor miRNAs in CRC, playing a role in the regulation of several cellular processes including proliferation, migration and chemoresistance [18,19,20]. According to the literature data, we confirmed the suppressor role of these two miRs, demonstrating their involvement in the RAS–MAPK axis and in the c-MYC pathway. At last, we also demonstrated that the inhibition of miR-31-3p and the upregulation of miR-143 and miR-145 in two KRAS mutated colorectal cancer cell lines determine the significant reduction of cell proliferation and migration after treatment with cetuximab (both *p* < 0.05), suggesting a possible pharmaceutical target role of these miRs whose modulation could be used in the overcoming, also if partially, the anti-EGFR resistance in mCRC.

With the objective of understanding the molecular mechanisms underlying the c-MYC-induced resistance, we performed a microarray analysis that highlighted some significant downstream genes that could be specific druggable target. The analysis demonstrated that the anti-EGFR resistance involved some genes belonging to the cell cycle, to the apoptosis and to signal transduction pathways (about 30%, 20% and 20% respectively). Among these, Cyclin B1 and CRCND2 were significantly altered. High cyclin B1 and CRCND2 levels play a role in the alteration of the cell cycle control, in the progression and in the higher metastatic capacity in CRC [27,28]. Conversely, CDKN1B, a cyclin-dependent kinase inhibitor, is frequently down-regulated in CRC, with an aggressive tumor behavior and a poor clinical outcome [29]. On the contrary, other pathways, such as the apoptotic, signal transduction and cell growth and proliferation pathways and the DNA repair, RNA processing and binding factors pathways showed relatively reduced alterations. Anyhow, some of these are directly involved in cetuximab resistance, such as UBE2C, and could be valid targets to overcome the EGFR-blockage resistance [30].

Finally, several papers have described the association between c-MYC gene function and its nuclear localization and, at the same time, the not correlation between c-MYC protein nuclear expression and mRNA expression, given the tight regulation of c-MYC protein levels [31]. In this sense, the immunohistochemical analysis, which has a significant correlation with the nuclear protein expression of c-MYC (nuclear fraction), represents an excellent and easily feasible method for a functional evaluation of c-MYC expression.

## 4. Material and Methods

### 4.1. Clinical Features

Clinical records of patients affected by mCRC and treated at our institution with anti-EGFR antibodies between May 2009 and April 2016 were reviewed. The study was carried out in accordance with the Declaration of Helsinki and consent for chemotherapy was obtained by all patients, also including the consent for retrospective analysis of all clinical data, according to the Ethical Committee of the Catholic University School of Medicine (Roma PROT. OM-2009-1 and 200603000). At the beginning of treatment with anti-EGFR all patients were in good physical condition with Performance Status zero. Patients were excluded in case of any previous treatment for metastatic disease, in case of known extra-hepatic disease at the time of diagnosis or in case of other cancers diagnosed within the previous 5 years. Objective response was assessed using RECIST 1.1 criteria to define complete response (CR), partial response (PR), stable disease (SD) and progressive disease (PD) [32].

The objectives of the study were to validate known mechanisms and identify novel drivers of response/resistance to anti-EGFR treatment in colorectal cancer. In this study we follow the REMARK criteria to individuate a new biomarker [33].

Eligibility criteria included anti-EGFR treatment, availability of stored tissue sample enough for quality-controlled molecular analysis, evaluation of response according to RECIST criteria, no serious concomitant illness that could have affected treatment duration or survival. Other criteria for eligibility were age 18 and 75 years, and one measurable tumor. Included patients also had Eastern Cooperative Oncology Group performance status of 0 to 2, life expectancy >3 months, and adequate hematologic, hepatic, and renal function. Patients who had suspected brain metastases or other cancers within the previous 5 years were also excluded. Only patients, who were given computerized tomography at regular intervals, not longer than three months, were considered. The study was retrospectively carried out on tissue samples belonging to 121 patients (Table 1 and Figure 1) with mCRC whose histological diagnosis was performed on biopsies or surgical resection tissues. One hundred and twenty-one patients were rule-based on the KRAS, NRAS and BRAF wild type and received Cetuximab as anti-EGFR antibody (only two patients received cetuximab in monotherapy) in the first-line therapy. Treatment associated with cetuximab (initial dose of 400 mg/m^2^ and then 250 mg/m^2^ weekly) included FOLFOX-6 (oxaliplatin 100 mg/m^2^ and folinic acid 400 mg/m^2^ on day 1 followed by a 5-FU bolus 400 mg/m^2^ and a 46-h infusion of 2400 mg/m^2^) or FOLFIRI (irinotecan 180 mg/m^2^ and folinic acid 400 mg/m^2^ on day 1 followed by a 5-FU bolus 400 mg/m^2^ and a 46-h infusion of 2400 mg/m^2^). Only five (4%) patients had a skin toxicity grade 3 and after a cetuximab dose modification they continued anti-EGFR therapy at full dose (Table 1).

In addition, the analysis was performed on metastatic liver tissues belonging to 33 out of 121 (27%) patients (Table 1 and Figure 1), considered technically unresectable at diagnosis and with liver only colorectal cancer metastases (LOM), subject to conversion chemotherapy (chemotherapy plus cetuximab as above described) that led to surgical treatment. The metastatic samples eligibility criteria included: histologically confirmed CRC, liver only measurable (according to RECIST 1.1 criteria) metastases considered technically non-resectable by the local multi-disciplinary team (MTD), no serious concomitant illness (uncontrolled hypertension, recent myocardial infarction, unstable angina, heart disease grade ≥2 according to NYHA criteria, uncontrolled diabetes, renal or liver failure), that could have affected treatment duration, survival or the possibility of surgery at the time of diagnosis. Patients had to be diagnosed to have LOM of colorectal cancer based on a contrast-enhanced computed tomography (CT-scan) or magnetic resonance imaging (MRI). Patients were considered unresectable if metastases involved all hepatic veins, both portal branches or hepatic arteries, irrespective of size and number of lesions. Unresectable were also considered those patients who had disseminated bilobar metastases preventing from complete R0 resection. Therapeutic decisions concerning each individual patient were taken by the same MTD, which included two liver-dedicated surgeons, two medical oncologists, a radiologist, an interventional radiologist and a pathologist. Only patients who received CT-scan or MRI and consequent MTD evaluation at diagnosis and at regular intervals not longer than three months were included. Liver resection was considered feasible when a R0 resection was technically possible with a residual healthy liver enough to assure an adequate function. Surgery was attempted whenever a disease-free margin was technically achievable. Liver resection was defined as R1 when microscopic margin involvement was demonstrated, while it was considered R2 when macroscopic disease was not removed. Surgery had to be performed within 8 weeks from the last chemotherapy dose.

The study also included 45 KRAS, NRAS mutated patients (Table S1 and Figure 1) that were not eligible for anti-EGFRs containing regimens. These patients were treated with anti-angiogenic drug bevacizumab plus chemotherapy: the FOLFOXIRI-bevacizumab combination. Bevacizumab was given as a 5 mg/kg intravenous dose. FOLFOXIRI consisted of a 165 mg/m^2^ intravenous infusion of irinotecan for 60 min, followed by an 85 mg/m^2^ intravenous infusion of oxaliplatin given concurrently with 200 mg/m^2^ leucovorin for 120 min, followed by a 3200 mg/m^2^ continuous infusion of fluorouracil for 48 h.

Progression-free survival (PFS) was defined as the time from cetuximab or other chemotherapy treatment initiation to the date of first documented progression, radiologically or clinically. Overall survival (OS) was defined as the time from cetuximab or other chemotherapy to the date of death due to any cause or last day of follow-up. Patients who had not progressed or died were censored at the date of last follow-up.

### 4.2. Immunohistochemistry for c-MYC and Staining Evaluation Score

Formalin-fixed, paraffin embedded sections (3 μm thick) were mounted on positive charged glass slides. For antigen retrieval to detect c-MYC protein, deparaffinized and rehydrated sections were boiled in TRIS-EDTA buffer solution (pH 9; for Abcam antibody) for 20 min or microwaved for 8–15 min in 10 mM sodium citrate (pH 6.0; for Santa Cruz Biotechnology antibody). The slides were cooled, and endogenous peroxidase were blocked with peroxidase block buffer (citric acid 0.04 M, Na_2_HPO4 × 2H_2_O 0.12 M, NaN3 0.03 M and H_2_O_2_ at 1.5% *v*/*v*) for 15 min at room temperature. Then, the sections were blocked with 1% BSA in PBS for 20 min and incubated for 72 h at 4 °C with rabbit monoclonal antibody anti-c-MYC (clone Y69; 1:100 diluition, Abcam, Cambridge, UK).

The primary antibodies were visualized using the avidin-biotin-peroxidase complex method (UltraTek HRP Anti-polyvalent, ScyTek, Logan, UT, USA) according to the instruction manual. 3,3′-diaminobenzidine was used as the enzyme substrate to observe the specific antibody localization, and Mayer hematoxylin was used as a nuclear counterstain.

The staining intensity of tissue slides was evaluated independently by 2 observers (L.M.L and M.M.) who were blinded toward the patients’ characteristics and survival. Cases with disagreement were discussed using a multiheaded microscope until agreement was achieved. To assess differences in staining intensity, an immunoreactivity scoring system was applied. c-MYC expression in each specimen was scored according to the extent (percent of stained cells) and intensity of nuclear expression staining. The score for the extent of the immunohistochemistry (IHC) stained area was scaled as 0 for no IHC signal at all, 1 for 1–30%, 2 for 31–70% and 3 for 71–100% of tumor cells stained. The score for IHC intensity was also scaled as 0 for no IHC signal, 1 for weak, 2 for moderate, and 3 for strong IHC signals. The final score used in the analysis was calculated by multiplying the extent score and intensity score, with a maximum score equal to 9.

The selection of cutoff scores for c-MYC expression in the prediction of anti-EGFR therapy response were based on ROC analysis Receiver Operating Characteristics (ROC) curve analysis. At each score, the sensitivity and specificity values were plotted thus generating a ROC curve. The score located closest to the point with both maximum sensitivity and specificity on the curve (0.0, 1.0), was selected as the cutoff score leading to the greatest number of tumors which were correctly classified as having or not having the outcome. Area under the ROC curves summarize the discriminatory power of c-MYC expression for the outcome with values of 0.5 indicating low power and those closer to 1.0 higher power. We compare three different immunohistochemical score for low and high expression of c-MYC: 0–1 versus 2–9; 0–3 versus 4–9; 0–6 versus 7–9. The immunohistochemical score 0–3 and 4–9, discriminating samples with low or high expression c-MYC, respectively, was identified as the best cutoff score in the prediction of anti-EGFR therapy response (*p* < 0.001; AUC = 0.899; 95% CI from 0.832 to 0.946; Youden index J = 0.6204; Sensitivity 96%; Specificity 66%; Figure S4). c-MYC staining that was confined to some nuclei of scattered cells at the bases of crypts (<5% positive cells) was considered as a normal mucosa staining pattern (internal positive control; 1–2 score). Negative controls were tumor sections stained in the absence of the primary antibody. Positive controls were Burkitt lymphoma samples. The c-MYC expression was also evaluated on a cohort of 40 normal colonic mucosae samples, without pathological features, belonging to the same mCRC cohort. All samples were stained more than once, and the results were highly reproducible [34].

### 4.3. DNA Extraction and KRAS, NRAS, BRAF Mutational Analysis

DNA was extracted from three 10 μm-slides from paraffin-embedded tissues using QIAamp DNA FFPE Tissue Kit (Qiagen, Milan, Italy), following the manufacturer’s protocol. In order to minimize contamination by normal cells, the tumor areas dissected for DNA and RNA extraction contained at least 70% of tumor cells. KRAS, NRAS and BRAF mutational analysis were carried out by pyrosequencing in all naïve mCRC and metastatic samples before and after TT treatment, as previously described [35]. RAS and BRAF mutations were performed using therascreen KRAS Pyro Kit, therascreen RAS Extension Pyro Kit and therascreen BRAF Pyro Kit (Qiagen). Mutation status was determined by pyrosequencing on the Qiagen PyroMark Q24.

### 4.4. The HER2 and c-MET Gene Amplification

c-MET gene copy numbers were assessed by fluorescence in situ hybridization (FISH) using ZytoLight SPEC MET/CEN7 Dual Color Probe (Zytovision, Bremerhafen, Germany). Before hybridization, sections were deparaffinized, dehydrated and immersed in citrate buffer (Merck KGaA, Darmstadt, Germany) pH 6 at 98 °C for 15 min, and subsequently washed twice in distilled water for 2 min. The sections were air dried and pretreated with pepsin for 5 min before denatured for 10 min at 75 °C. After hybridization at 37 °C for 20 h, slides were washed and counterstained with 1.5 μg/mL 4′,6′-diamidino-2-phenylindole (DAPI) mounting medium (Vectashield, Vector Laboratories, Burlingame, CA, USA) and coverslips were fixed with nail polish. For each probe, the numbers of c-MET and CEN7 per nuclei were separately scored and mean cMET/CEN7 ratio was determined. FISH MET gene amplification was defined as FISH positive, when MET/CEP7 ratio was >2.2 or small gene clusters (≥4 copies) independent of the MET to CEP 7 ratio.

HER2 amplification was performed using the INFORM HER2/neu Dual ISH DNA Probe Cocktail assay (Ventana Medical Systems, Inc, Tucson, AZ, USA). The DISH assay was performed according to the manufacturer’s recommended protocol for surgical specimens. The HER2/neu (black) to chromosome enumeration probe 17 (CEP17) (red) ratio was manually counted using a light microscope and the result was confirmed by a second investigator. At least 20 cells were counted. The criteria consist of a combination of the HER2/CEP17 ratio and the average number of HER2 signals per cell. The HER2 gene amplification was scored as “amplified” if the case had a HER2/CEP17 signal count ratio of 2.0 or if the HER2/CEP17 signal count ratio was <2.0 but the average number of HER2 signals per cell was 6.0. A score of “equivocal” was given if the case had a HER2/CEP17 signal count ratio of <2.0 and the average number of HER2 signals per cell was ≥4.0 and <6.0. A score of “not amplified” was given if the case had a HER2/CEP17 signal count ratio of <2.0 and the average number of HER2 signals was <4.0.

### 4.5. RNA Extraction and Real Time Analysis

RNA was extracted from three 10 μm-slides from paraffin-embedded tissues using RNeasy FFPE Kit or miRNeasy Mini Kit (Qiagen), following the manufacturer’s protocol. Real time PCR was performed using the KAPA SYBR FAST One-Step qRT-PCR Kit (KAPA-Biosystems, Boston, MA, USA), or QuantiFast Multiplex RT-PCR Master Mix (Qiagen), following the manufacturer’s protocol, in CFX96™ Real-Time PCR Detection Systems (Bio-Rad, Rome, Italy) [36]. Briefly, RNA was added (25 ng) to the mixture containing 10 μL of KAPA SYBR FAST qRT-PCR Master Mix (2X), 0.4 μL the forward primer and the reverse primer (10 μM), 0.4 μL of dUTP (10 mM) and 0.4 μL of KAPA RT Mix (50×) to a final volume of 20 μL. The amplification conditions were: an initial cycle at 42 °C for 5 min, a denaturation step at 95 °C for 5 min, followed by 38 cycles at 95 °C for 5 s and 60 °C for 20 s. Each analysis was performed in duplicate and, for each gene, the expression level was normalized with the amount of β-actin (Table S5).

For real-time PCR of miRNAs, RNA (100 ng) was reverted by ImProm-II™ Reverse Transcription System (Promega, Madison, WI, USA) and then amplified in real time by the Maxima SYBR Green qPCR Master Mix (2×) (Fermentas, Milan, Italy). Briefly, 12.5 μL of Maxima SYBR Green qPCR Master Mix 2× were added to 0.3 μM of forward and reverse primer, 2 μL of cDNA and water to a final volume of 25 μL. The amplification condition was: an initial denaturation step at 95 °C for 10 min, followed by 40 cycles at 95 °C for 15 s and 60 °C for 60 s. Normal colonic mucosae, without pathological features, of the same mCRC patients analyzed was considered as normal control. Each analysis was performed in duplicate and, the gene expression level was normalized with the amount of mirU6. All used primers and product lengths are shown in Table S5. Specific quantification of expression level of miRNA hsa-miR-31-3p was performed using specific TaqMan pre-designed assays on retrotranscribed RNA and an ABI 7900 HT Real-Time PCR System (assay ID 002113). Expression levels were normalized with the amount of mirU6 through the ΔΔCt method.

### 4.6. Gene Expression Array

Gene expression profile of c-MYC targets gene in naïve mCRC and in metastatic samples after TT showing high expression of c-MYC was compared to that of normal colonic mucosa and tumor samples that had a c-MYC low expression. Total RNA (1 mg) was reverted with RT^2^ First Strand cDNA Kit (SABiosciences, Qiagen, Milan, Italy) according to the manufacturer’s protocol and the array was performed using RT^2^ Profiler™ PCR Array Human MYC Targets (PAHS-177ZD, purchased from SABiosciences). For real-time PCR, first-strand cDNAs were added to the RT qPCR Master Mix (SABiosciences). Samples were heated for 10 min at 95 °C and then subjected to 40 cycles of denaturation at 95 °C for 15 s and annealing and elongation at 60 °C for 1 min. Data analysis was assessed using the RT2 Profiler PCR array data analysis template v3.0 (SABiosciences). Relative changes in gene expression were calculated using ΔΔCt (cycle threshold) method. The gene expression analysis was performed twice [37]. The most significant results obtained in RT2 PCR array were validated by real time PCR (primers listed in Table S5).

### 4.7. Cell Lines, Cell Cultures and Transfection

CRC cell lines HCT-116 and SW480 from ATCRC were cultivated in the recommended media (see www.atcc.org for details). Plasmid constructs, lentivirus infection and cell lines transduction with pLTR or its derivative pLTR143-5 were performed as previously described [19].

Anti-miRNA 31 inhibitor (anti-miR-31) (Applied Biosystems, Foster City, CA, USA) or Anti-miR negative control (Applied Biosystems) were transfected in CRC cell lines HCT-116 at a final concentration of 100 nM using the siPORT™ NeoFX™ Transfection Agent (Applied Biosystems) according to the manufacture’s protocol.

Cetuximab, an anti-EGFR human-mouse chimeric mAb was kindly provided by Merck Serono (Rome, Italy). The drug was dissolved in sterile dimethylsulfoxide (DMSO) and a 10 mmol/L working solution was prepared and stored in aliquots at −20 °C. Working concentrations were diluted in culture medium just before each experiment (final concentration was 1 μg/mL).

### 4.8. Cytotoxicity and Migration Assay

Cell lines were plated at a density of 2 × 10^4^/mL in 96-well plates in triplicate. Cytotoxicity assay was performed by using the CellTiter-Blue Viability Assay (Promega, Milan, Italy) as previously described [19]. Twenty-four hours after seeding, the cell lines were treated with the cetuximab addiction and the assay was performed after 72 h of treatment. The motility of transduced cell lines was evaluated 48 h post-sorting in 24-well transwell chambers (Corning Life Sciences, Corning, NY, USA).

### 4.9. Western-Blot Analysis for c-Myc

Briefly, cell samples were incubated in hypotonic lysis buffer (10 mM Hepes, pH 7.5, 10 mM KCl, 3 mM NaCl, 3 mM MgCl_2_, 1 mM EDTA, 1 mM EGTA) without detergent to swell up the cells. The plasma membrane of the swollen cells was then lysed by adding nonionic, nondenaturing detergent (NP-40, 0.5%) and the nuclei are pelleted. Cytoplasmic fraction (supernatant) is collected, and both the cytoplasmic fraction and the nuclei are denatured in sample buffer (Tris-HCl [pH 6.8], 10% SDS, 36% glycerine, 5% 2-mercaptoethanol, 0.03% bromophenol blue), boiled for 5 min, and then separated on SDS–polyacrylamide gel electrophoresis (SDS-PAGE). We used the Novex Sharp Pre-stained Protein Standard (Invitrogen, Milan, Italy) as a protein marker. Gels were blotted with transfer buffer (30 mM Tris, 240 mM glycine, 20% methanol) directly on pure nitrocellulose membrane (Bio-Rad) at 330 mA for 1 h. Blots were probed with rabbit monoclonal anti-c-MYC (clone Y69; 1:500 diluition, Abcam), rabbit polyclonal anti-E2F2 (1:500 diluition, Abcam), or with a mouse monoclonal anti-actin (Ab-5, 1:5000; BD Biosciences, Milan Italy) in TBST with gentle shaking. After membrane incubation with goat antimouse HRP-conjugated antiserum (1:1000; BD Biosciences) or with goat antirabbit HRP-conjugated antiserum (1:1000; BD Biosciences) in TBST for 1 h at room temperature with gentle shaking, blots were covered with enhancing chemiluminescence solution (GE Healthcare, UK) for 1 min and exposed to Kodak XAR-5 x-ray film (Kodak, Rochester, NY, USA) for 5 min. The c-Myc precipitates were subjected to densitometric analysis by using the Gel-Doc 2000 Quantity One program (Bio-Rad) or ImageJ software (NIH), after normalization with the actin intensity [38]. Raw images of the western blots with the protein marker is shown in Figure S5.

### 4.10. Statistical Analysis

Statistical analysis was performed using GraphPad-Prism 5 software (Graph Pad Software, San Diego, CA, USA) and MedCalc version 10.2.0.0 (MedCalc Software, Mariakerke, Belgium). Statistical comparison of continuous variables was performed by the Mann-Whitney U-test (*t* test), as appropriate. Comparison of categorical variables was performed by chi-square statistic, using the Fisher’s exact test. Kaplan-Meier survival curves were plotted and differences in survival between groups of patients were compared using the log-rank test. Multivariate analysis was performed using the Cox proportional hazards regression analysis including only those clinical and biological variables with a *p*-value of 0.10 or lower on univariate analysis. *p*-values less than 0.05 were considered as statistically significant. In the figures, if a *p*-value is less than 0.05, it is flagged with one star (*); if a *p*-value is less than 0.01, it is flagged with two stars (**); if a *p*-value is less than 0.001, it is flagged with three stars (***).

## 5. Conclusions

Although other confirmatory studies are necessary, our retrospective monocentric report demonstrated, that HEM is a predictive and a prognostic marker for the anti-EGFR plus chemotherapy treated mCRC, identifying a subgroup of patients where the effector mechanism of c-MYC is already active, probably determined by an already present kinome reprogramming phenomena, finally providing other potential therapeutic targets to overcome the TT resistance.

## Figures and Tables

**Table 1:** Clinical features of anti-EGFR cohort.

Patient Clinical Features	n° Cases (Percentage)
Sex	
-male	71 (59%)
-female	50 (41%)
Age (yrs)	
-median (range)	60 (19–75)
-mean ± sd	62 ± 10.59
Tumor location	
-cecum-ascending	30 (25%)
-transverse	6 (5%)
-descending	3 (2%)
-sigma-rectum	82 (68%)
Histotypes	
-well to moderate	83 (69%)
-poorly	29 (24%)
-mucinous	9 (7%)
ECOG PS	
-0	67 (55%)
-1	49 (40%)
-2	5 (4%)
Metastases	
-one or two sites	115 (95%)
-confined to liver	37 (31%)
Worst skin toxicity seen	
-0	15 (12%)
-1	70 (58%)
-2	31 (26%)
-3	5 (4%)
-4	0
Metastases after TT	
-resected	33 (27%)
-not resected	88 (73%)
c-MYC expression	
-HME	45 (37%)
-LME	76 (63%)

## Supplementary Materials

The following are available online at https://www.mdpi.com/2072-6694/12/3/638/s1, Figure S1: Panel A and B. Kaplan-Meier curves for PFS and OS of RAS-BRAF mutated mCRC patients treated with antiangiogenetic plus chemotherapy stratified by c-MYC expression. LME patients (blue-line) was not significantly associated to a better PFS (*p* = 0.7159) and OS (*p* = 0.8083) respect to HME (red-line). Figure S2: The figure shows the not significant correlation between c-MYC immunohistochemical expression score and c-Myc RNA expression (Spearman r = 0.13; *p* = 0.4289). Figure S3: Panel A. Kaplan-Meier curves for PFS of RAS-BRAF wild-type anti-EGFR mCRC patients stratified by miR-31-3p expression (65 patients). LME patients (blue-line) was significantly associated to a better PFS (*p* = 0.0017) respect to HME (red-line); Panel B. The figure shows the significant correlation between c-MYC immunohistochemical expression score and miR-31-3p expression (65 patients; Spearman r = 0.66; *p* < 0.0001). Figure S4: Receiver operating characteristic (ROC) curves for c-MYC immunohistochemical score (0–3 score versus 4–9 score) and PFS. Figure S5: Raw images of the western blots with the protein marker. Table S1: Clinical features of anti-angiogenetic plus chemotherapy treated cohort. Table S2: Multivariate analysis of PFS. Table S3: Multivariate analysis of OS. Table S4: real-time analysis of altered genes. Table S5: Primers utilized in RT-PCR analysis.

## Abbreviations

mCRC	metastatic colorectal cancer
TT	targeted therapy
LOM	only colorectal cancer metastases
MTD	multi-disciplinary team
CT-scan	contrast-enhanced computed tomography
MRI	magnetic resonance imaging
HME	high c-MYC expression
LME	low c-MYC expression
PFS	progression free survival
OS	overall survival
miR	miRNA
NCM	normal colonic mucosa
