# Supplementary material for: c-MYC Expression Is a Possible Keystone in the Colorectal Cancer Resistance to EGFR Inhibitors
Source: Cancers (Basel). 2020 Mar 10;12(3):638. doi: 10.3390/cancers12030638 (PMC7139615; doi:10.3390/cancers12030638)
Supplement: Supplementary file 1 [file cancers-12-00638-s001.zip › Supplementary Figures.pdf]

Supplementary Materials

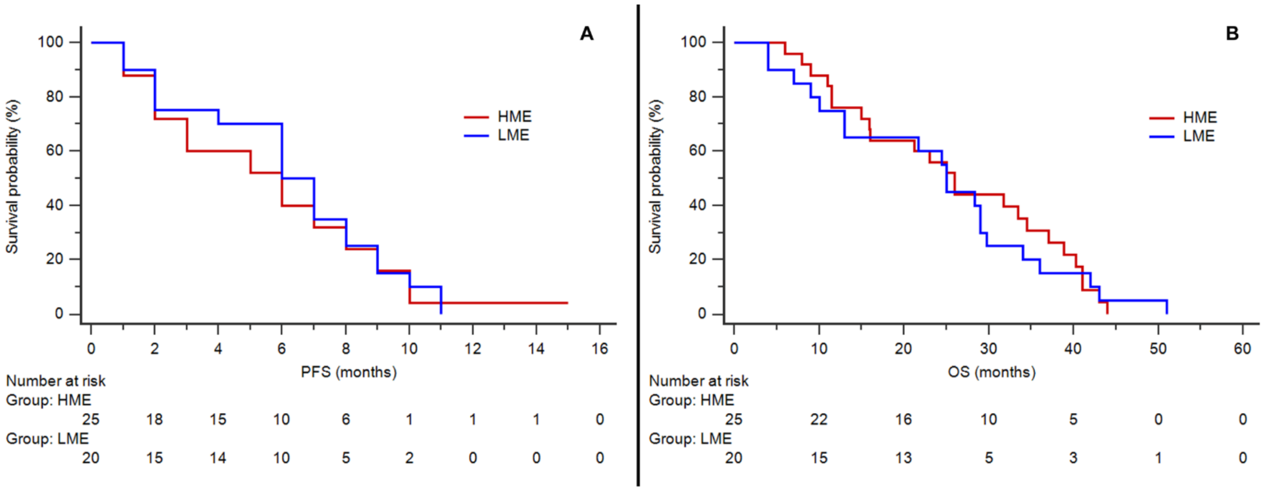

Figure S1: Panel A and B. Kaplan-Meier curves for PFS and OS of RAS-BRAF mutated mCRC patients treated with antiangiogenetic plus chemotherapy stratified by c-MYC expression. LME patients (blue-line) was not significantly associated to a better PFS (p = 0.7159) and OS (p = 0.8083) respect to HME (red-line).

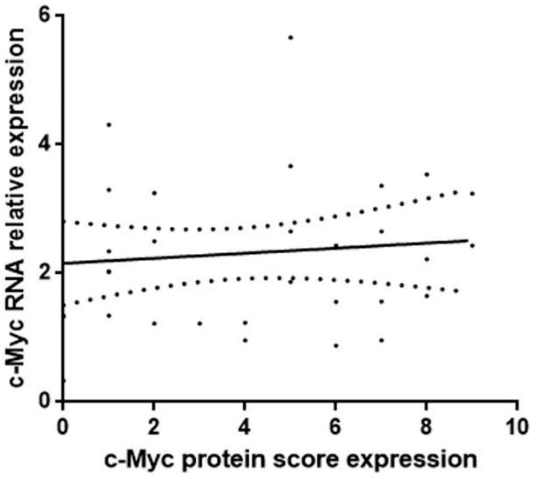

Figure S2: The figure shows the not significant correlation between c-MYC immunohistochemical expression score and c-Myc RNA expression (Spearman r = 0.13; p = 0.4289).

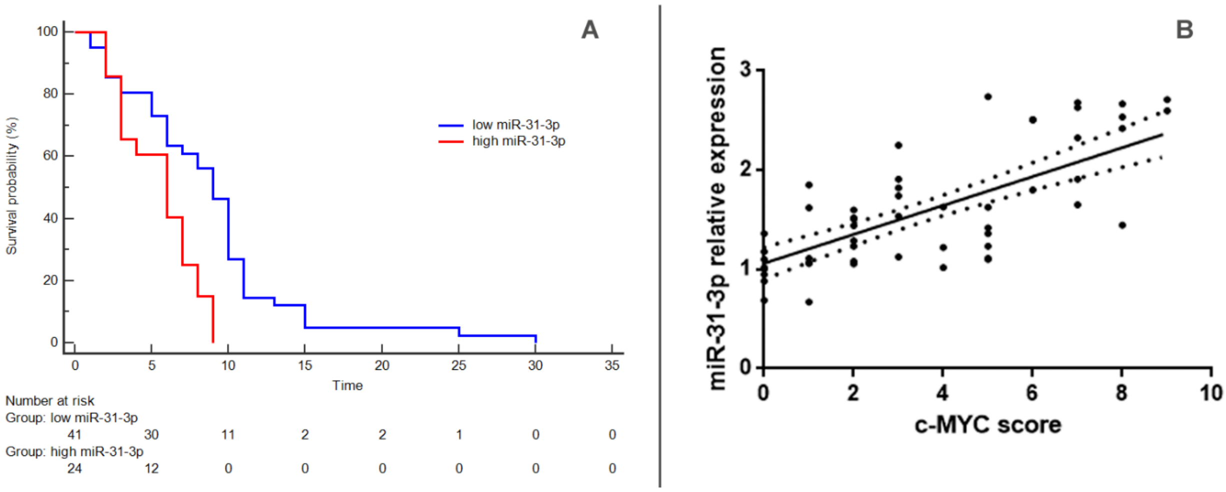

Figure S3: Panel A. Kaplan-Meier curves for PFS of RAS-BRAF wild-type anti-EGFR mCRC patients stratified by miR-31-3p expression (65 patients). LME patients (blue-line) was significantly associated to a better PFS (p = 0.0017) respect to HME (red-line); Panel B. The figure shows the significant correlation between c-MYC immunohistochemical expression score and miR-31-3p expression (65 patients; Spearman r = 0.66; p < 0.0001).

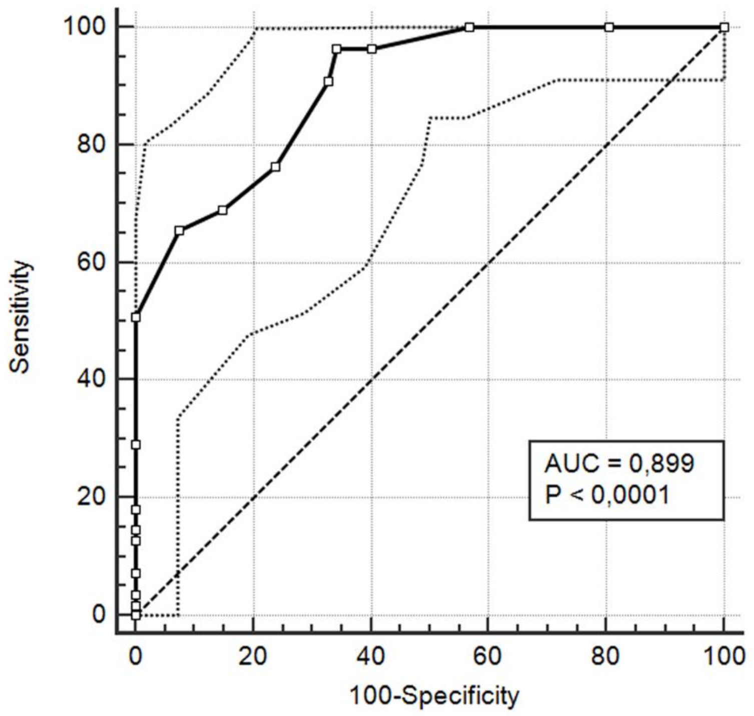

Figure S4: Receiver operating characteristic (ROC) curves for c-MYC immunohistochemical score (0–3 score versus 4–9 score) and PFS.

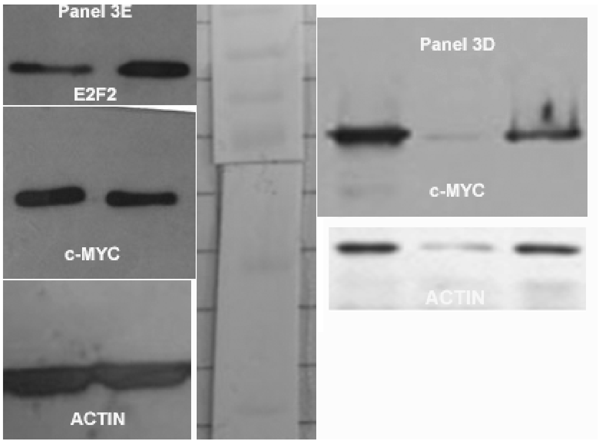

Figure S5: Raw images of the western blots with the protein marker.
